# Fatal septicemia in a patient with cerebral lymphoma and an Amplatzer septal occluder: a case report

**DOI:** 10.1186/1752-1947-5-554

**Published:** 2011-11-24

**Authors:** Claudia Stöllberger, Adam Bastovansky, Josef Finsterer

**Affiliations:** 1 Krankenanstalt Rudolfstiftung, Juchgasse 25, A-1030 Wien, Austria; 2 Danube University Krems, Doktor-Karl-Dorrek-Straße 30, A-3500 Krems, Austria

## Abstract

**Introduction:**

The Amplatzer septal occluder is frequently used for percutaneous closure of an atrial septal defect. Complications include thrombosis and embolism, dislocation, cardiac perforation, and, rarely, infection. We report the case of a patient who had survived an occluder-related thromboembolism two years previously.

**Case presentation:**

A 72-year-old Caucasian woman had received a septal occluder because of an atrial septal defect seven years ago. Two years ago, she underwent chemotherapy of a non-Hodgkin lymphoma, developed atrial fibrillation, and experienced a left-sided occluder thrombosis with stroke and peripheral embolism. Now, she presented with cerebral lymphoma, received glucocorticoids, and subsequently developed skin lesions. Swabs from the lesions and blood cultures were positive for methicillin-resistant *Staphylococcus aureus *and *Pseudomonas aeruginosa*. Endocarditis, however, was considered only two months later and echocardiography suggested aortic valve endocarditis. Despite antibiotic therapy, she died three days later because of septicemia, and no post-mortem investigation was carried out. It remains uncertain whether the septal occluder was endothelialized or infected and whether explantation might have changed the outcome.

**Conclusions:**

If infections occur in patients with a septal occluder, endocarditis should be considered and echocardiography should be performed early. To prevent a fatal outcome, explantation of the septal occluder should be considered, especially in patients with problems that suggest delayed endothelialization. Post-mortem investigations, including bacteriologic studies, should be carried out in patients with a septal occluder in order to assess the focal and global long-term effects of these devices.

## Introduction

The Amplatzer septal occluder (SO) (AGA Medical Corporation, Plymouth, MN, USA) is a frequently used device for percutaneous closure of an atrial septal defect (ASD). Complications of occluders include thrombosis and embolism, dislocation, cardiac perforation, and, rarely, infection [1-5]. We report the fatal course of septicemia in a patient who had already survived an SO-related thromboembolism two years previously [6].

## Case presentation

Our patient was a 72-year-old Caucasian woman who had a hemodynamically relevant ASD and who at the age of 65 years had received a 22 mm SO because of increasing exertional dyspnea. She did not complain of arrhythmia, and the results of a coronary angiography were normal. The further course was complicated by a non-Hodgkin lymphoma and probably by chemotherapy-induced Evans syndrome. Symptomatic atrial fibrillation was diagnosed 58 months after implantation, and a therapy with bisoprolol and acetylsalicylic acid was started. Between 56 and 59 months after implantation, a left-sided SO thrombosis developed, as demonstrated by computed tomography [6]. The SO thrombosis led to ischemic stroke and peripheral embolism, necessitating surgical embolectomy in all extremities and oral anticoagulation (OAC) with phenprocoumon. Complete disappearance of the SO thrombus was demonstrated by transesophageal echocardiography five months later. OAC was continued because of atrial fibrillation and a serological indication for hypercoagulability (lupus anticoagulants, elevation of homocysteine, and factor VIII).

**Figure 1:**
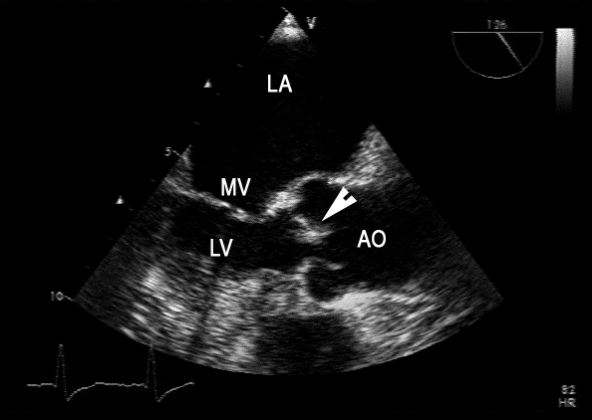
**A transesophageal echocardiogram shows the left atrium (LA), parts of the mitral valve (MV), the left ventricle (LV), the ascending aorta (AO), and a thickened aortic cusp (arrow), the last of which is highly suggestive of endocarditis**.

At the age of 72 years, vertigo, headache, and visual field defects occurred. Despite normal results of a cerebrospinal fluid examination, relapsing lymphoma was suspected on the basis of cerebral magnetic resonance imaging (MRI) findings. Oncologists prescribed dexamethasone 32 mg/day. Symptoms regressed and MRI findings improved. Four weeks after the initiation of glucocorticoids, excoriations on both legs developed and antibiotic therapy with cefazolin 6 g/day was given for 10 days. Two weeks later, fever occurred, and sulbactam/ampicillin 3 g/day was given for nine days. Glucocorticoid therapy was continued. Swabs taken from the excoriations and one of five blood cultures were positive for methicillin-resistant *Staphylococcus aureus *(MRSA). *Pseudomonas aeruginosa *grew on a further excoriation swab. Since MRSA was found in only one blood culture, prolonged antibiotic therapy was deemed not to be indicated. Unfortunately, endocarditis was not considered, and she was discharged without echocardiography. Four weeks later, she fell, developed a hematoma, and was re-hospitalized because of a hemorrhagic erysipela. MRSA grew on a swab from the erysipela. *P. aeruginosa *grew in blood cultures and on excoriation swabs of the legs. Linezolid 1200 mg/day and sulbactam/ampicillin 3 g/day were started. This time, endocarditis was considered, and transesophageal echocardiography showed no thrombus or vegetations on the SO but was highly suggestive of an aortic valve vegetation (Figure 1). Our patient died three days later because of septicemia and multi-organ failure. An autopsy was not performed.

## Discussion

The pathogenesis can be explained as follows: Skin lesions developed as a side effect of glucocorticoids, and this also favored immunosuppression. The infectious agents that lastly caused septicemia, multi-organ failure, death, and probably endocarditis either entered via the skin or may have derived from the SO, although the evidence for the latter assumption is lacking.

Thrombus formation and infection are rare complications of ASD occluders, and only six reports of device-related infection have been published to date [1-5]. Both thrombi and vegetations on occluders present on echocardiography as shaggy masses typically with multiple mobile strands. Although in our patient these findings were not found on echocardiography, the SO might have served as a nidus for the bacteria. This hypothesis is substantiated by histological findings of explanted SOs, showing a chronic inflammatory reaction inside the occluder [7]. In our patient, who already had experienced an unusually late SO thrombosis, delayed endothelialization, probably induced by chemotherapy or glucocorticoids, and thus propensity for bacterial colonization might have occurred. Unfortunately, these considerations are only speculative since no pathologic examination or post-mortem cultures of the device have been carried out. Had echocardiography been performed earlier and the aortic valve vegetations detected, cardiac surgery, including inspection and eventually removal of the SO, would have been a therapeutic option. Such a procedure has been chosen in reported cases of endocarditis after occluder implantation [1-5,7].

## Conclusions

This case shows that, in patients with SO and infections, clinicians should have a high suspicion for endocarditis. Echocardiography should be performed early and repeated if there is bacteremia. To prevent a fatal outcome, explantation of the SO should be considered, especially in patients who already had SO-related problems that suggested delayed endothelialization. Post-mortem investigations, including bacteriologic studies, should be carried out in patients with SO in order to assess the focal and global long-term effects of these devices.

## Abbreviations

ASD: atrial septal defect; SO: septal occluder; MRI: magnetic resonance imaging; MRSA: methicillin-resistant *Staphylococcus aureus*; OAC: oral anticoagulation.

## Consent

Written informed consent was obtained from the patient's relatives for publication of this case report and accompanying images. A copy of the written consent is available for review by the Editor-in-Chief of this journal.

## Competing interests

The authors declare that they have no competing interests.

## Authors' contributions

CS analyzed and interpreted the patient data regarding the course of the disease and wrote the manuscript. AB performed the radiological studies. JF performed the neurological investigations and was a major contributor in writing the manuscript. All authors read and approved the final manuscript.
